# Effect of Patient’s Characteristics and Surgical Technique on the Patient Outcomes and Satisfaction After Endoscopic Lumbar Discectomy—A Long-Term Retrospective Study

**DOI:** 10.3390/jcm14051411

**Published:** 2025-02-20

**Authors:** Youssef Jamaleddine, Ahmad Haj Hussein, Ahmad Afyouni, Zaid Mayta, Lemir Majed El Ayoubi, Pascale Salameh, Ramzi Moucharafieh, Mohamad Omar Honeine, Mohammad Badra

**Affiliations:** 1 Gilbert and Rose-Marie Chagoury School of Medicine, Lebanese American University, Beirut 0379, Lebanon; youssefjd@outlook.com (Y.J.); ahmad.hajhussein@lau.edu (A.H.H.); pascalesalameh1@hotmail.com (P.S.); omar.hnieneh.oh@gmail.com (M.O.H.); 2 Faculty of Medical Sciences, Lebanese University, Hadath 1533, Lebanon; ahmad.afyouni@st.ul.edu.lb (A.A.); le75@aub.edu.lb (L.M.E.A.); 3 Faculty of Medicine, Beirut Arab University, Beirut 1107, Lebanon; zaidmayta11@gmail.com; 4 Department of Neurology, American University of Beirut Medical Center, Beirut 1107, Lebanon; 5 Faculty of Pharmacy, Lebanese University, Hadath 1533, Lebanon; 6 Institut National de Santé Publique d’Épidémiologie Clinique et de Toxicologie-Liban (INSPECT-LB), Beirut 1103, Lebanon; 7 Department of Orthopedic Surgery, Faculty of Medicine, Balamand University, Beirut 1300, Lebanon; ramzi.moucharafieh@fty.balamand.edu.lb; 8 Department of Orthopedics and Traumatology, Clemenceau Medical Center, Beirut P.O. Box 11-2555, Lebanon

**Keywords:** disc, endoscopic, discectomy, interlaminar, transforaminal, satisfaction, outcomes

## Abstract

**Background**: Percutaneous endoscopic lumbar discectomy (PELD) is a minimally invasive surgical technique for the treatment of lumbar disc herniation. Despite its growing popularity, limited research has explored the influence of patient characteristics and the choice of technique on post-operative outcomes and patient satisfaction. **Objective**: To investigate the impact of patient characteristics and surgical technique (interlaminar vs. transforaminal) on the surgical outcomes and patient satisfaction following PELD. **Methods**: A retrospective analysis was conducted on 177 patients who underwent PELD (53.1% males, age = 46.11 ± 14.2 years), including 147 patients with the interlaminar approach and 30 with the transforaminal approach. Demographic data, pre-operative clinical features, surgical technique, intra-operative and post-operative complications and complaints, patient-reported outcomes (disability, quality of life, satisfaction), and revision surgery rates were documented and analyzed. The mean follow-up duration was 5.55 years ± 2.73 years. **Results**: No significant differences were observed in demographics, pre-operative status, or post-operative complaints and complication rates between two surgical techniques, except that transforaminal technique showed a higher incidence for dural tear and persistent muscle weakness (*p* = 0.028 and *p* = 0.046, respectively). Both techniques led to excellent patient-reported outcomes with no significant differences. Total patient satisfaction with PELD was 93.8%, which correlated positively with the absence of complications and complaints and negatively with persistent back pain, recurrent herniation and revision surgery. **Conclusions**: Interlaminar and transforaminal PELD are both effective and safe minimally invasive surgical techniques for the treatment of lumbar disc herniation with a high patient satisfaction rate. Further prospective studies are warranted to confirm these findings.

## 1. Introduction

Lumbar spine disc herniation is the leading cause of lower back pain worldwide [1]. As ageing occurs, a degenerative process leading to disc herniation most commonly affects the lumbar spine, followed by the cervical spine [2]. Depending on the extent to which the nervous system is compressed, disc herniation can emerge in various presentations such as back pain, neurogenic claudication, and radiculopathy including weakness and numbness impacting different body areas [3,4,5]. The high prevalence and treatment costs of the disease have attracted medical personnel to undergo extensive research to attain the most favourable therapeutic approach [1].

Disc herniation management depends on the extent of the nervous system involvement and its impact on the patient’s daily life activities. Taking into consideration the level, location, and severity of disc herniation, mild cases are usually treated by medications and lifestyle modification. In the case of more severe cases or the failure of conservative treatment, surgical interventions are often required to improve the patient’s quality of life [6]. Nevertheless, the surgical management of disc disease showed more rapid relief from back pain symptoms when compared to conservative management [7]. This is especially true in short-term relief; however, the long-term effects are still controversial [8].

Open lumbar discectomy (OLD) remained the gold standard through the mid-1990s, up until the introduction of the endoscopic technique, which showed an improvement in the overall clinical outcomes [9,10,11]. Clinical investigations have recently focused on the minimally invasive percutaneous endoscopic lumbar discectomy (PELD), showing a good outcome in properly selected cases, depending on the level, location, and morphology of the herniated disc [12]. PELD has gained great interest due to its minimal invasiveness, short hospital stay, fast recovery, and impressive clinical outcomes both on short- and long-term follow-up [13]. More specifically, percutaneous interlaminar endoscopic discectomy (PIED) is known to be advantageous over percutaneous transforaminal endoscopic discectomy (PTED) in treating the disc herniation of lower levels due to the wider intervertebral interlaminar space [13,14].

In the literature, there is limited research available exploring the effect of patient characteristics on post-operative outcomes and patient satisfaction using PELD comparing interlaminar and transforaminal techniques. Our study aims to assess post-operative outcomes and the satisfaction rate of PELD in both techniques. We believe that patients undergoing PELD will exhibit a high satisfaction rate and a minimal incidence of complications post-operatively, with no significant differences between the techniques used.

## 2. Materials and Methods

### 2.1. Study Design

A retrospective study was conducted by patient self-reported information via phone calls and data extraction from medical records. A questionnaire was developed, including multiple scales and personalized questions investigating the impact of patient characteristics and endoscopic discectomy techniques on post-operative outcomes and patient satisfaction.

### 2.2. Participants and Indication of Surgery (Figure 1)

Patients eligible for participation in this study were those who had undergone endoscopic lumbar discectomy for disc herniation at Clemenceau Medical Center (CMC) between 2013 and 2022. Inclusion criteria comprised individuals aged 18 years or above, who had undergone endoscopic discectomy surgery for lumbar disc herniation, with the surgery performed at least one year before data collection. To note, all surgeries were performed by the same senior surgeon (MB).

Exclusion criteria comprised patients who had other types of discectomies, those who had non-lumbar endoscopic discectomy, individuals who had the operation less than a year before data collection, those with multi-level disc herniation, revision disc surgeries, and patients with missing data.

Patients underwent conservative treatment, including medications (NSAIDs, analgesics, pregabalin/gabapentin), physiotherapy, and potentially epidural injections. Endoscopic discectomy was considered and indicated for those with persistent pain or motor weakness despite conservative treatment.

**Figure 1:**
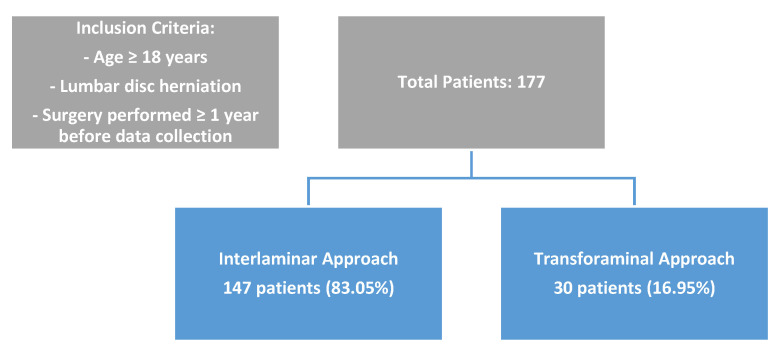
Participant flowchart.

### 2.3. Ethical Considerations

This research is a retrospective study that was conducted respecting the ethical principles following the declaration of Helsinki developed originally in 1964. Confidentiality and anonymity of all data and participants were assured. Institutional Review Board (IRB) approval from the ethical committee of Clemenceau Medical Center was acquired (Ref: ERRC/RMRR/03/2023). Participants agreed to an informed consent to confirm their willingness to participate in this study with guarantees that their anonymity would be preserved.

### 2.4. Data Collection and Tools Used

Data were collected from patients’ medical records, operative reports, endoscopic videos, and self-reported information via phone calls. Data collected included sociodemographic, clinical, and surgical characteristics. Sociodemographic and clinical characteristics were taken pre-operatively. Sociodemographic characteristics included age, gender, marital status, residential region, profession, and education level. Clinical characteristics included body mass index (BMI), smoking status, alcohol consumption, the presence of neurologic motor deficit (defined as the MRC scale less than 5 over 5), the presence of chronic diseases, and the duration of symptoms before surgery. Surgical characteristics included disc location and herniation level, previous steroid injections, surgical technique, and intraoperative complications (dural tear and nerve injury). Furthermore, data regarding clinical outcomes such as post-operative complications (bacterial infection, bleeding, paralysis, recurrent herniation, persistent back pain, persistent muscle weakness, and leg dysesthesia), reoperation, quality of life, disability levels, and patient satisfaction were collected.

The quality of life was assessed post-operatively at the time of data collection, using the Short Form Health Survey (SF-12), an abbreviated version of the SF-36 [15], and it consists of 12 items. The SF-12 survey yields two scores for the patient’s quality of life: the Physical Component Score (PCS) and Mental Component Score (MCS). These are calculated by multiplying weighted scores by the answers of each item, summing them up, and adding a constant, to generate a mean of 50 and a standard deviation of 10 in the general population [16]. Scores above 50 indicate above-average health-related quality of life, whereas scores below 50 indicate below-average health.

Disability levels were assessed, post-operatively at the time of data collection, using the Oswestry Disability Index (ODI), a tool designed to quantify disability specifically related to low back pain. The ODI comprises 10 sections: pain intensity, personal care, lifting, walking, sitting, standing, sleeping, sex life, social life, and travelling. Each section consists of six statements, with each statement scored from 0 to 5. To calculate the index, scores from all sections are summed and then multiplied by two. The resulting index ranges from 0 to 100, where a score of 0–20 is considered minimal disability, 21–40 moderate disability, 41–60 severe disability, 61–80 crippled, and 81–100 bed-bound [17].

Patient satisfaction was assessed, post-operatively at the time of data collection, using a 1-item Likert scale that is scored from 0 to 4 ranging from very dissatisfied to very satisfied.

Nearly all patient data, including characteristics, history, and follow-up information, were meticulously documented in electronic medical records, significantly reducing the risk of recall bias.

To prevent missing data, our team ensured all patients completed the scale questionnaire during data collection, resulting in a complete dataset.

### 2.5. Statistical Method

The normality of the data was assessed using the Shapiro test. The *t*-test and Mann–Whitney U test were used as appropriate to compare the mean of the dependent variable between two categories. Chi-square was used for the bivariate analyses of the categorical variables, with the technique used (transforaminal or interlaminar) as the independent variable. The correlation between two quantitative variables was assessed using the Pearson correlation test. Ordinal logistic regression was used as well. The *p*-value < 0.05 was considered statistically significant.

All statistical analyses were performed using IBM SPSS Statistics for Windows, Version 27.0. Armonk, NY, USA: IBM Corp.

## 3. Results

Our final sample size consisted of a total of 177 patients (53.1% males), with a mean age of 46.11 ± 14.2 years. The Interlaminar technique was used in 147 (83.05%) patients, and transforaminal technique was used in 30 (16.95%) patients. No significant differences were found between the two surgical techniques in the demographic characteristics including gender, age, BMI, marital status, governorate, educational level, type of work, smoking habits, and alcohol intake (Table 1).

The majority of patients with the interlaminar technique had a herniated disc at the L5-S1 level (85; 57.8%), paracentral location (145; 98.6%), and with no neurological deficit (88; 59.8%), while the majority of patients with the transforaminal technique had a herniated disc at the L4-L5 level (23; 76.6%), paracentral location (21; 70%), with pre-operative neurological deficit (18; 60%) (*p* < 0.001, *p* < 0.001, and *p* = 0.046, respectively). At the time of data collection, the mean duration since surgery was 5.43 ± 2.81 years and 6.17 ± 2.27 years in patients undergoing PLED, the interlaminar and transforaminal techniques, respectively, without a significant difference (*p* = 0.179), Overall, the mean duration since surgery for all the patients was 5.55 ± 2.73 years (Table 2).

During the surgery, patients with the interlaminar technique had no dural tear, while two patients (6.7%) with the transforaminal technique had a dural tear (*p* = 0.028), and no patients with the interlaminar technique suffered from a nerve injury, while one patient (3.3%) suffered from a nerve injury in those with the transforaminal technique with no significant difference between techniques (*p* = 0.169). As for the complaints and complications following the surgery, no significant differences were found between the two techniques except for persistent muscle weakness, where the interlaminar technique (3.4%) had a lower rate compared to the transforaminal technique (13.3%) (*p* = 0.046). Rates of bacterial infection, bleeding, and paralysis were 0% in both techniques. In terms of revision surgeries for the same herniated disc, 5.4% of patients with the interlaminar technique underwent another surgery, while a higher rate (10%) of those with the transforaminal technique underwent another surgery with no significant differences (*p* = 0.346), and all revision surgeries were for reherniation of the disc. Importantly, the disc reherniation rate was also higher in the transforaminal group (13.3%) compared to the interlaminar group (7.5%), although this difference was not statistically significant (*p* = 0.289). Overall, the disc reherniation rate was 8.5% (Table 3).

At the time of follow-up, the disability levels of the patients were very low, where only 8 (4.5%) had severe disability and 3 (1.7%) were crippled. Physical and Mental Component Scores of the SF-12 scales were 48.16 ± 9.84 and 51.71 ± 9.71, respectively. A total of 166 (93.8%) patients were satisfied or very satisfied by the outcome of the endoscopic discectomy surgery, and only 5 (2.8%) patients were dissatisfied or very dissatisfied. No significant differences were found between interlaminar and transforaminal techniques regarding ODI, PCS, MCS, or satisfaction (Table 4).

Table 5 shows the satisfaction rate across patients’ characteristics. Patients who were very dissatisfied, dissatisfied, and neither satisfied nor dissatisfied were grouped as not satisfied or neutral. Patients who were satisfied and very satisfied were grouped as satisfied. No significant differences were seen between the satisfaction rates across patients’ demographics.

A lower satisfaction rate was associated with recurrent disc herniations (80% vs. 95.1%, *p* = 0.021), undergoing revision surgery (81.8% vs. 94.58%, *p* < 0.001) and persistent back pain (50% vs. 95.8%, *p* < 0.001). On the other hand, patients with no complications or complaints reported significantly higher satisfaction (97.8% vs. 80%, *p* < 0.001).

Satisfied patients following the endoscopic surgery had significantly better physical health (48.98 ± 9.40 vs. 35.83 ± 8.23, *p* < 0.001), better mental health (52.22 ± 9.45 vs. 43.94 ± 10.79, *p* = 0.011), and lower disability levels (13.08 ± 13.79 vs. 43.94 ± 10.79, *p* < 0.001) when compared to not satisfied patients.

Bivariate correlations are presented in Table 6. Satisfaction was negatively correlated with the ODI score (r = −0.469, *p* < 0.001). On the other hand, satisfaction was positively correlated with the Physical Component Score (r = 0.402, *p* < 0.001) and Mental Component Score (r = 0.205, *p* < 0.01).

Table 7 presents the odds ratio of ordinal logistic regression analysis, aimed at identifying the variables associated with the satisfaction of patients regarding the surgery. The model had a Nagelkerke pseudo R^2^ of 0.372, thus explaining 37.2% of the satisfaction distribution. The analysis showed that the odds of being more satisfied were significantly higher among patients of higher age (OR = 1.036, *p* < 0.05), with higher education (OR = 19.921, *p* < 0.05). On the other hand, the analysis showed that the odds of being dissatisfied with the surgery were significantly higher in patients who had more previous steroid injections (OR = 0.235, *p* < 0.05) and complications after the surgery (OR = 0.238, *p* < 0.05).

## 4. Discussion

More than 95% of lumbar disc herniation occurs at the level of L4-L5 and L5-S1 [1]. Currently, the tendency of lower back surgery is shifting towards using PELD instead of OLD [12], with the specific PELD technique varying between the morphology of the herniated disc. To our knowledge, this is the first study in the Middle East that comparatively analyzed the clinical outcomes and satisfaction rates of PLED and its different techniques (PTED vs. PIED) in the treatment of lower back pain, aiming to offer a reliable reference for improving patient outcomes.

This study observed no significant differences in demographic characteristics between patients undergoing PIED and PTED, indicating the feasibility of both techniques across diverse patient demographics and populations. As for the clinical characteristics of our sample, notable disparities were found in the distribution of herniated disc level, herniated disc location, and the presence of neurological deficits between the two techniques. In our sample, PIED was chosen more frequently for the L5-S1 level due to the technical challenges associated with accessing the disc through the foramen. The L5-S1 level poses technical difficulties such as the presence of a high iliac crest, the large transverse process of L5, and the potential for hidden disc materials. In such cases, PIED is often considered a more suitable alternative [13,18]. Of the patients who had PIED, 98.6% had their disc herniation in the paracentral region, in comparison to 70% in PTED, with the remaining cases divided equally between foraminal and central regions.

As for the post-operative complications and complaints, the interlaminar approach demonstrated a lower incidence of dural tears (0%) compared to the transforaminal approach (6.7%). This difference may be attributed to differences in anatomical access between the two techniques. Both techniques resulted in almost no nerve injury (only one PTED case), without any case of bleeding, paralysis, or bacterial infection, in addition to comparable rates of post-operative complications and complaints, except for persistent muscle weakness, which was significantly lower in the PIED group (3.4% vs. 13.3%). Further investigation into the factors contributing to this difference is warranted. No significant differences were found in the rates of revision surgeries and disc reherniation between both techniques. Similar findings have been reported in other studies, which observed comparable clinical outcomes between the two techniques, although the interlaminar approach demonstrated a slight advantage in terms of radiation exposure, operative time, and complications [19,20].

Patients reported outcomes that were similar in both techniques used. Although there is no baseline ODI and SF-12 scores to compare with, most of the two samples had minimal-to-moderate disability levels post-operatively based on the ODI score. No significant differences were found between ODI, PCS, and MCS scores (*p* = 0.34, *p* = 0.594, *p* = 0.879, respectively). These results align with the results of other similar studies [21,22,23].

Patients showed a high satisfaction rate of 93.9% and 93.3% with PIED and PTED, respectively, with no significant differences between both, underscoring the effectiveness of the two techniques in alleviating symptoms and improving quality of life.

Patients with recurrent disc herniation, persistent back pain, and revision surgeries were significantly less satisfied, but patients who did not have complications or complaints reported significantly higher satisfaction. Consistent with expectations, satisfaction was significantly and negatively correlated with the post-operative ODI score but significantly and positively correlated with PCS and MCS. In the regression model built for predicting satisfaction, the odds of the patients being satisfied increase with older age and higher education but decrease in patients who had complications, three or more previous steroid injections, and increased disability post-operatively. Gender and intraoperative dural tears did not significantly impact patient satisfaction post-surgery.

PELD was associated with a higher patient satisfaction rate and lower complication rate when compared with OLD [24]. Our study also reflected this trend, with a satisfaction rate of approximately 93.8%, higher than that of OLD that ranged from 70 to 95% [25], and of microdiscectomy that scored 80% [26]. As for the disability rate, the majority of our patients showed low ODI scores. Similarly, PED reported a significantly lower ODI after surgery when comparing microscope-assisted tubular discectomy and open microdiscectomy [27,28]. However, in a systematic review and meta-analysis comparing the clinical outcomes of minimally invasive discectomy with the OLD and microdiscectomy, there was no difference in the ODI between the three techniques [29]. This highlights the need for further prospective research to investigate the potential reasons for this heterogeneity.

Our results showed an overall 8.5% reherniation rate for PELD. Comparatively, a systematic review reported reherniation rates ranging from 1% to 12% for OLD, 1% to 10.8% for microendoscopic discectomy, and 5.5% to 9.6% for PELD [30]. Our findings align with the higher end of the range observed in the systematic review, suggesting a slightly elevated risk of reherniation associated with PELD in our study population.

A total of 6.2% of patients included in our study required revision surgery. In another study comparing PELD and OLD, PELD showed a higher reoperation rate when compared with OLD, of 5.38% and 2.28%, respectively [31,32]. The reoperation rate after microdiscectomy is considerably higher than that of other surgical techniques. Two different studies reported that 25% and 18.5% of patients have reoperated after index microdiscectomy [31,32].

One study showed that the incidence of infection after endoscopic spine surgery ranges between 0.1% and 0.4% [13]. However, another study compared OLD with PELD and showed lower incidences of infections with 0.83% in the endoscopic surgery group and 1.18% in the open surgery group [33]. Interestingly, none of the patients (0%) in our sample experienced post-operative infections, which is consistent with the finding of another study [34]. This highlights the relative safety of the PELD procedure. In our sample, prophylactic antibiotics included Cefuroxime pre-operatively, with Clindamycin administered for patients with known allergies. Additionally, all patients underwent scrubbing and draping in a sterile manner to minimize the risk of infection.

Another systematic review and meta-analysis compared the clinical efficacy between PELD and posterior open lumbar microdiscectomy (OLMD) for the treatment of symptomatic lumbar disc herniation. Consistent with our findings, neither wound infection nor epidural hematoma was observed in the PELD group, which might indicate that PELD has the advantage of less surgical trauma compared with OLMD [35].

Our study identified an intraoperative dural tear rate of 1.1%, similar to the rates of other studies on PELD [36] but lower than that of microdiscectomy procedures (2.85%) [37]. In one study, open discectomy exhibited a dural injury rate of 1.4%, while microdiscectomy showed a higher incidence of 3.3% [38], both higher than PELD, indicating the superiority of the endoscopic approach in terms of minimizing dural tears. The lower incidence of dural tears in endoscopic discectomy compared to open microdiscectomy is likely due to several factors. High-definition cameras provide magnified and clear views of the surgical field, enabling surgeons to precisely identify and avoid the dura mater. Furthermore, endoscopic instruments are designed for precision and allow for the targeted removal of the herniated disc material with minimal manipulation of surrounding structures, thereby reducing the risk of dural injury. Regarding post-operative leg dysesthesia, our findings showed a rate of 10.2%, which is less than another study that reported a leg dysesthesia rate of 14.29% [39]. Leg dysesthesia in our sample did not significantly differ based on the surgical technique used. Additionally, satisfaction levels were not significantly affected by the presence of leg dysesthesia. Further research into risk factors and the long-term impact of dysesthesia on quality of life could provide valuable insights into the clinical relevance of this finding.

## 5. Conclusions

This study underscores the effectiveness, safety, and high patient satisfaction of percutaneous endoscopic lumbar discectomy (PELD) in treating lumbar disc herniation. Clinical outcomes from both interlaminar and transforaminal methods were favourable, with a high satisfaction rate and few complications. These findings confirm the reliability of PELD as a minimally invasive alternative to traditional open discectomy, with a personalized surgical approach enhancing patient outcomes. To further validate these results and improve patient care, future multicenter, prospective studies are recommended, including subgroup analyses based on factors such as age or the severity of lumbar disc disease. Additionally, exploring the use of emerging technologies, such as artificial intelligence, navigation or 3D printing, for pre-operative planning and surgical approach personalization could provide valuable insights for optimizing PELD procedures.

## 6. Limitations

Despite the numerous strengths of our study, it also presents some limitations. The retrospective design of our study with recall bias, no baseline data on patient’s ODI, PCS, and MCS, conducted at a single medical centre, makes it hard to generalize the results on the whole population. Furthermore, this study may not have fully accounted for confounding variables such as surgeon experience and pre-operative rehabilitation. Future research should focus on prospective, multicenter studies with larger sample sizes and comprehensive data collection methods to overcome these limitations to provide more robust evidence regarding the comparative effectiveness of interlaminar and transforaminal techniques in lumbar endoscopic discectomy, with the aim of ensuring that the findings are representative of the general population with lumbar disc disease.

## Figures and Tables

**Table 1:** Demographic characteristics of the participants across the technique used for PED.

		Technique *N* (%)		Total (*N* = 177)	*p*-Value
		Interlaminar (*n* = 147)	Transforaminal (*n* = 30)		
Age	Mean ± *SD*	46.61 ± 14.4	44.63 ± 13.29	46.11 ± 14.2	0.534
Gender	Male	79 (53.7%)	15 (50%)	94 (53.1%)	0.708
	Female	68 (46.3%)	15 (50%)	83 (46.9%)	
BMI	Mean ± *SD*	27.48 ± 4.65	28.76 ± 4.31	27.7 ± 4.61	0.166
Marital Status	Married	119 (81%)	25 (83.3%)	144 (81.4%)	0.760
	Unmarried	28 (19%)	5 (16.7%)	33 (18.6%)	
Governorate	Beirut	70 (47.6%)	20 (66.7%)	90 (50.8%)	0.288
	Mount Lebanon	38 (25.9%)	7 (23.3%)	45 (25.4%)	
	North Lebanon/Akkar	8 (5.4%)	1 (3.3%)	9 (5.1%)	
	South Lebanon/Nabatieh	22 (15%)	1 (3.3%)	23 (13%)	
	Bekaa/Baalbek-Hermel	9 (6.1%)	1 (3.3%)	10 (5.6%)	
Education	Elementary School	17 (11.6%)	6 (20%)	23 (13%)	0.535
	Baccalaureate	40 (27.2%)	8 (26.7%)	48 (27.1%)	
	Bachelor’s Degree	60 (40.8%)	11 (36.7%)	71 (40.1%)	
	Master’s Degree	21 (14.3%)	2 (6.7%)	23 (13%)	
	PhD/MD	9 (6.1%)	3 (10%)	12 (6.8%)	
Profession	Does Not Work	51 (34.7%)	9 (30%)	60 (33.9%)	0.468
	Healthcare Profession	10 (6.8%)	4 (13.3%)	14 (7.9%)	
	Outside Healthcare	86 (58.5%)	17 (56.7%)	103 (58.2%)	
Smoker	No	52 (35.4%)	13 (43.3%)	65 (36.7%)	0.410
	Yes	95 (64.6%)	17 (56.7%)	112 (63.3%)	
Alcoholic	No	110 (74.8%)	21 (70%)	131 (74%)	0.792
	Yes, Occasionally	31 (21.1%)	8 (26.7%)	39 (22%)	
	Yes, Regularly	6 (4.1%)	1 (3.3%)	7 (4%)	

**Table 2:** Clinical characteristics of the participants across the technique used for PED.

		Technique *N* (%)		Total (*N* = 177)	*p*-Value
		Interlaminar (*n* = 147)	Transforaminal (*n* = 30)		
Herniated Disc Level	L2–L3	1 (0.7%)	2 (6.7%)	3 (1.7%)	<0.001
	L3–L4	4 (2.7%)	4 (13.3%)	8 (4.5%)	
	L4–L5	57 (38.8%)	23 (76.7%)	80 (45.2%)	
	L5–S1	85 (57.8%)	1 (3.3%)	86 (48.6%)	
Herniated Disc Location	Foraminal	1 (0.7%)	4 (13.3%)	5 (2.8%)	<0.001
	Extraforaminal	0 (0%)	1 (3.3%)	1 (0.6%)	
	Paracentral	145 (98.6%)	21 (70.0%)	166 (93.8%)	
	Central	1 (0.7%)	4 (13.3%)	5 (2.8%)	
Neurological (Motor) Deficit	No	88 (59.9%)	12 (40%)	100 (56.5%)	0.046
	Yes	59 (40.1%)	18 (60%)	77 (43.5%)	
Duration After Surgery (Years)	Mean ± *SD*	5.43 ± 2.81	6.17 ± 2.27	5.55 ± 2.73	0.179
Duration of Symptoms Before Surgery	<1 Month	9 (6.1%)	3 (10%)	12 (6.8%)	0.546
	1–6 Months	55 (37.4%)	10 (33.3%)	65 (36.7%)	
	6–12 Months	27 (18.4%)	8 (26.7%)	35 (19.8%)	
	1–2 Years	20 (13.6%)	5 (16.7%)	25 (14.1%)	
	>2 Years	36 (24.5%)	4 (13.3%)	40 (22.6%)	
Previous Steroid Injection	No	97 (66%)	19 (63.3%)	116 (65.5%)	0.835
	1 Time	16 (10.9%)	4 (13.3%)	20 (11.3%)	
	2 Times	17 (11.6%)	2 (6.7%)	19 (10.7%)	
	3 Times	5 (3.4%)	1 (3.3%)	6 (3.4%)	
	>3 Times	12 (8.2%)	4 (13.3%)	16 (9%)	

**Table 3:** Post-operative complications and complaints of the participants across the technique used for PED.

		Technique *N* (%)		Total (*N* = 177)	*p*-Value
		Interlamina (*n* = 147)	Transforaminal (*n* = 30)		
Dural tear	No	147 (100%)	28 (93.3%)	175 (98.9%)	0.028
	Yes	0 (0%)	2 (6.7%)	2 (1.1%)	
Nerve injury	No	147 (100%)	29 (96.7%)	176 (99.4%)	0.169
	Yes	0 (0%)	1 (3.3%)	1 (0.6%)	
Complications	No Complications or Complaints	114 (77.6%)	23 (76.7%)	137 (77.4%)	0.916
	Bacterial Infection	0 (0%)	0 (0%)	0 (0%)	-
	Bleeding	0 (0%)	0 (0%)	0 (0%)	-
	Paralysis	0 (0%)	0 (0%)	0 (0%)	-
	Recurrent Herniation	11 (7.5%)	4 (13.3%)	15 (8.5%)	0.289
	Persistent Back Pain	7 (4.8%)	1 (3.3%)	8 (4.5%)	1.000
	Persistent Muscle Weakness	5 (3.4%)	4 (13.3%)	9 (5.1%)	0.046
	Leg Dysesthesia	16 (10.9%)	2 (6.7%)	18 (10.2%)	0.742
Revision Surgery	No	139 (94.6%)	27 (90%)	166 (93.8%)	0.346
	Yes	8 (5.4%)	3 (10%)	11 (6.2%)	

**Table 4:** Patients’ reported outcomes across the technique used for PED.

		Technique *N* (%)		Total (*N* = 177)	*p*-Value
		Interlaminar (*n* = 147)	Transforaminal (*n* = 30)		
Disability (ODI)	Minimal Disability	111 (75.5%)	21 (70%)	132 (74.6%)	0.340
	Moderate Disability	26 (17.7%)	8 (26.7%)	34 (19.2%)	
	Severe Disability	8 (5.4%)	0 (0%)	8 (4.5%)	
	Crippled	2 (1.4%)	1 (3.3%)	3 (1.7%)	
	Bed-Ridden	0 (0%)	0 (0%)	0 (0%)	
PCS	Mean ± *SD*	48.34 ± 9.88	47.28 ± 9.77	48.16 ± 9.84	0.594
MCS	Mean ± *SD*	51.65 ± 9.73	51.95 ± 9.77	51.71 ± 9.71	0.879
Satisfaction	Very Dissatisfied	3 (2%)	0 (0%)	3 (1.7%)	0.616
	Dissatisfied	2 (1.4%)	0 (0%)	2 (1.1%)	
	Neither Satisfied nor Dissatisfied	4 (2.7%)	2 (6.7%)	6 (3.4%)	
	Satisfied	22 (15%)	6 (20%)	28 (15.8%)	
	Very Satisfied	116 (78.9%)	22 (73.3%)	138 (78%)	

PCS: Physical Component Score; and MCS: Mental Component Score.

**Table 5:** Clinical and demographical characteristics across the satisfaction rate for PED.

		Satisfaction *N* (%)		Total (*N* = 177)	*p*-Value
		Not Satisfied or Neutral (*n* = 11)	Satisfied (*n* = 166)		
Age (years)	Mean ± *SD*	44.09 ± 16.50	46.24 ± 14.08	46.11 ± 14.20	0.527
Gender	Male	4 (4.2%)	90 (95.8%)	94 (100%)	0.251
	Female	7 (8.4%)	76 (91.6%)	83 (100%)	
Marital status	Married	9 (6.25%)	135 (93.75%)	144 (100%)	0.968
	Unmarried	2 (6%)	31 (94%)	33 (100%)	
BMI	Mean ± *SD*	26.69 ± 4.07	27.76 ± 4.65	27.70 ± 4.61	0.562
Duration after Surgery	Mean ± *SD*	4.82 ± 2.22	5.6 ± 2.76	5.55 ± 2.73	0.434
Governorates	Beirut	9 (10%)	81 (90%)	90 (100%)	0.187
	Mount Lebanon	0 (0%)	45 (100%)	45 (100%)	
	North Lebanon/Akkar	0 (0%)	9 (100%)	9 (100%)	
	South Lebanon/Nabatieh	1 (4.3%)	22 (95.7%)	23 (100%)	
	Bekaa/Baalbek-Hermel	1 (10%)	9 (90%)	10 (100%)	
Educational level	Elementary School	4 (17.4%)	19 (82.6%)	23 (100%)	0.088
	Baccalaureate	4 (8.33%)	44 (91.67%)	48 (100%)	
	Bachelor’s Degree	3 (4.2%)	68 (95.8%)	71 (100%)	
	Master’s Degree	0 (0%)	23 (100%)	23 (100%)	
	PhD/MD	0 (0%)	12 (100%)	12 (100%)	
Profession	Does Not Work	5 (8.33%)	55 (91.67%)	60 (100%)	0.492
	Healthcare Profession	0 (0%)	14 (100%)	14 (100%)	
	Outside Healthcare	6 (5.8%)	97 (94.2%)	103 (100%)	
Smoking	No	3 (4.6%)	62 (95.4%)	65 (100%)	0.502
	Yes	8 (7.1%)	104 (92.9%)	112 (100%)	
Alcoholism	No	9 (6.8%)	122 (93.2%)	131 (100%)	0.726
	Yes, Occasionally	2 (5.12%)	37 (94.88%)	39 (100%)	
	Yes, Regularly	0 (0%)	7 (100%)	7 (100%)	
Herniated Disc Level	L2-L3	0 (0%)	3 (100%)	3 (100%)	0.794
	L3-L4	1 (12.5%)	7 (87.5%)	8 (100%)	
	L4-L5	4 (5%)	76 (95%)	80 (100%)	
	L5-S1	6 (7%)	80 (93%)	86 (100%)	
Herniated Disc Location	Foraminal	0 (0%)	5 (100%)	5 (100%)	0.855
	Extraforaminal	0 (0%)	1 (100%)	1 (100%)	
	Paracentral	11 (6.63%)	155 (93.37%)	166 (100%)	
	Central	0 (0%)	5 (100%)	5 (100%)	
Neurological (Motor) Deficit	No	5 (45.5%)	95 (57.2%)	100 (100%)	0.446
	Yes	6 (54.5%)	71 (42.8%)	77 (100%)	
Duration of Symptoms Before Surgery	<1 Month	2 (16.6%)	10 (83.4%)	12 (100%)	0.405
	1–6 Months	2 (3%)	63 (97%)	65 (100%)	
	6–12 Months	3 (8.5%)	32 (91.5%)	35 (100%)	
	1–2 Years	1 (4%)	24 (96%)	25 (100%)	
	>2 Years	3 (7.5%)	37 (92.5%)	40 (100%)	
Previous Steroid Injections	No	6 (5.17%)	110 (94.83%)	116 (100%)	0.383
	1 Time	2 (10%)	18 (90%)	20 (100%)	
	2 Times	0 (0%)	19 (100%)	19 (100%)	
	3 Times	1 (16.6%)	5 (83.4%)	6 (100%)	
	>3 Times	2 (12.5%)	14 (87.5%)	16 (100%)	
Technique Used	Interlaminar	9 (6.1%)	138 (93.9%)	147 (100%)	0.910
	Transforaminal	2 (6.7%)	28 (93.3%)	30 (100%)	
Dural Tear	No	11 (6.2%)	164 (93.8%)	175 (100%)	0.714
	Yes	0 (0%)	2 (100%)	2 (100%)	
Nerve Injury	No	11 (6.25%)	165 (93.75%)	176 (100%)	0.796
	Yes	0 (0%)	1 (100%)	1 (100%)	
Hypertension	No	10 (7.7%)	120 (92.3%)	130 (100%)	0.176
	Yes	1 (2.1%)	46 (97.9%)	47 (100%)	
Diabetes	No	10 (6.45%)	145 (93.55%)	155 (100%)	0.729
	Yes	1 (4.55%)	21 (95.45%)	22 (100%)	
Dyslipidemia	No	11 (8.14%)	124 (91.86%)	135 (100%)	0.056
	Yes	0 (0%)	42 (100%)	42 (100%)	
CVD	No	11 (7%)	146 (93%)	157 (100%)	0.222
	Yes	0 (0%)	20 (100%)	20 (100%)	
Cancer	No	11 (6.4%)	161 (93.6%)	172 (100%)	0.559
	Yes	0 (0%)	5 (100%)	5 (100%)	
Chronic Lung Disease	No	11 (6.5%)	158 (93.5%)	169 (100%)	0.456
	Yes	0 (0%)	8 (100%)	8 (100%)	
Renal Failure	No	11 (6.3%)	164 (93.7%)	175 (100%)	0.714
	Yes	0 (0%)	2 (100%)	2 (100%)	
Hypothyroidism	No	10 (6.1%)	155 (93.9%)	165 (100%)	0.753
	Yes	1 (8.4%)	11 (91.6%)	12 (100%)	
Hyperthyroidism	No	11 (6.3%)	162 (93.7%)	173 (100%)	0.603
	Yes	0 (0%)	4 (100%)	4 (100%)	
Recurrent Herniation	No	8 (4.9%)	154 (95.1%)	162 (100%)	0.021
	Yes	3 (20%)	12 (80%)	15 (100%)	
Persistent Back Pain	No	7 (4.2%)	162 (95.8%)	169 (100%)	<0.001
	Yes	4 (50%)	4 (50%)	8 (100%)	
Persistent Muscle Weakness	No	9 (5.3%)	159 (94.7%)	168 (100%)	0.410
	Yes	2 (23.3%)	7 (77.7%)	9 (100%)	
Leg Dysesthesia	No	9 (5.6%)	150 (94.4%)	159 (100%)	0.364
	Yes	2 (21.1%)	16 (88.9%)	18 (100%)	
No Complications	Complications	8 (20%)	32 (80%)	40 (100%)	<0.001
	No Complications	3 (2.2%)	134 (97.8%)	137 (100%)	
Revision Surgery	No	9 (5.42%)	157 (94.58%)	166 (100%)	<0.001
	Yes	2 (18.2%)	9 (81.8%)	11 (100%)	
PCS	Mean ± *SD*	35.83 ± 8.23	48.98 ± 9.40	48.16 ± 9.84	<0.001
MCS	Mean ± *SD*	43.94 ± 10.79	52.22 ± 9.45	51.71 ± 9.71	0.011
ODI Score	Mean ± *SD*	36 ± 18.46	13.08 ± 13.79	14.51 ± 15.11	<0.001

*N* = 177, BMI: body mass index; CVD: cardio-vascular disease; IBD: inflammatory bowel disease; PCS: Physical Component Score; MCS: Mental Component Score; ODI Score: Oswestry Disability Index Score; and SD: standard deviation.

**Table 6:** Bivariate correlations between patient outcomes.

	Satisfaction	ODI Score	Physical Component Score
Satisfaction	-		
ODI Score	−0.469 ***	-	
Physical Component Score	0.402 ***	−0.737 ***	-
Mental Component Score	0.205 **	−0.302 ***	0.158 *

N = 177; *** *p* < 0.001, ** *p* < 0.01, * *p* < 0.05; and ODI Score: Oswestry Disability Index Score.

**Table 7:** Ordinal logistic regression analysis of patient satisfaction.

		OR	95% CI		*p*-Value
			LL	UL	
Age		1.036	0.002	0.070	0.040
Gender	Female (ref)	-	-	-	-
	Male	0.568	−1.452	0.319	0.210
Education	Elementary School (ref)	-	-	-	-
	Baccalaureate	1.928	−0.573	1.886	0.295
	Bachelor’s Degree	9.224	0.811	3.632	0.002
	Master’s Degree	10.086	0.482	4.140	0.013
	PhD/MD	19.921	0.677	5.307	0.011
Previous Steroid Injection	No (ref)	-	-	-	-
	1 Time	0.855	−1.412	1.098	0.806
	2 Times	1.669	−1.013	2.038	0.510
	3 Times	0.039	−5.096	−1.417	<0.001
	>3 Times	0.235	−2.686	−0.213	0.022
Dural Tear	No (ref)	-	-	-	-
	Yes	0.116	−5.113	0.799	0.153
Reherniation	No (ref)	-	-	-	-
	Yes	4.456	−0.195	3.184	0.083
Complications/Complaints	No (ref)	-	-	-	-
	Yes	0.238	−2.545	−0.328	0.011
Duration After Surgery		0.968	−0.198	0.134	0.705
Physical Component Score		1.037	−0.022	0.094	0.227
Mental Component Score		1.024	−0.021	0.069	0.294
ODI Score		0.952	−0.090	−0.009	0.017

OR: odds ratio; 95% CI: 95% confidence interval; LL: lower limit; UL: upper limit; and ODI Score: Oswestry Disability Index Score.

## Data Availability

The data presented in this study are available upon request from the corresponding author due to restrictions (privacy).

## Abbreviations

The following abbreviations are used in this manuscript:

MDPI	Multidisciplinary Digital Publishing Institute
DOAJ	Directory of open access journals
TLA	Three letter acronyms
LD	Linear dichroism
OLD	Open lumbar discectomy
PELD	Percutaneous endoscopic lumbar discectomy
PIED	Percutaneous interlaminar endoscopic discectomy
PTED	Percutaneous transforaminal endoscopic discectomy
